# The impact of intelligent devices utilization on household medical expenditure of older adults in China

**DOI:** 10.1038/s41598-025-06627-0

**Published:** 2025-07-02

**Authors:** Jingjing Wang, Lianjie Wang

**Affiliations:** 1 School of Public Administration, Zhejiang University of Finance and Economics, Hangzhou, China; 2 Department of Sociology, Jiangnan University, Wuxi, China

**Keywords:** Older adults, Intelligent devices utilization, Family medical expenses, Healthy China, Health behaviors, Health care economics, Health services, Public health

## Abstract

With the rapid development of artificial intelligence, there is an increasing utilization of intelligent devices by older adults. The relationship between the utilization of intelligent devices and household medical expenditure has garnered widespread attention in academic circles. This paper employs data from the 2020 China Longitudinal Aging Social Survey (CLASS) to investigate the impact of intelligent device utilization by 9,718 older adults on household medical consumption. The research findings indicate that the utilization of intelligent devices significantly reduces household medical expenditure, and this conclusion remains valid after placebo tests and endogeneity treatments. From the perspective of heterogeneity, the impact of intelligent device utilization on household medical expenditure is more pronounced among higher age, those living with family, residents of eastern and western regions, and areas with high digital coverage. Quantile regression results reveal a “inverted U-shaped” trend in the impact of intelligent device utilization on household medical expenditure, with an initial increase followed by a decrease. Mechanism analysis suggests that intelligent device utilization reduces household medical expenditure by improving the health behaviors and decreasing the demand for medical services. Based on these findings, this paper argues that enterprises and research institutions should continue to develop intelligent devices tailored to the characteristics of older adults. The government should provide financial subsidies to purchase intelligent devices for older adults. By fully enhancing the utilization of intelligent devices in elderly health management, we can together provide a reference for controlling the excessive growth of medical expenses.

## Introduction

In recent years, healthy aging has become an important part of the social policies issued by the Chinese government. In 2016, the “Outline of the ‘Healthy China 2030’ Plan” emphasized the importance of developing new formats of health services, fostering a group of distinctive health management service industries, and exploring the advancement of intelligent health electronic products, wearable devices, and mobile application services for health and medical care. In 2024, the “Decision of the Central Committee of the Communist Party of China on Further Comprehensively Deepening Reform and Advancing Chinese-Style Modernization” pointed out the need to implement a health-prioritized development strategy and strengthen capabilities such as monitoring, early warning, and risk assessment. It can be seen that utilizing intelligent devices to actively conduct dynamic, comprehensive, and diversified health monitoring and management has become an important aspect of deeply implementing the health-prioritized development strategy. Continuous, real-time, and dynamic health monitoring and management through scientific methods align with the core concept of “prevention first” in the health-prioritized development strategy. At the same time, this also implies early detection and treatment of diseases to prevent minor illnesses from becoming major ones, thereby reducing medical costs and burdens on families and society. In addition, relevant studies have shown that the increase in medical expenses will exacerbate the debt burden of families and may even push families to the brink of bankruptcy^1^. Based on this, we hold that empowering health monitoring and dynamic management through digital-intelligence development, as well as providing personalized medical and healthcare services^2^ etc., are of great practical significance for curbing the rapid growth of medical expenses and enhancing the economic resilience of families.

The vigorous development of the digital economy and the rapid advancements in digital technologies such as artificial intelligence, the Internet of Things, and big data have provided excellent opportunities for the robust growth of intelligent devices. According to predictions by the International Data Corporation (IDC), in 2024, the global shipments of intelligent wearable devices will reach a staggering 559.7 million units. By the end of 2028, this shipment volume is expected to further increase to 645.7 million units, with a compound annual growth rate of 3.6%^3^. The utilization of intelligent devices offers possibilities for comprehensive and multi-dimensional health monitoring and management for older adults. Furthermore, with the rapid diversification of intelligent device types and the continuous enhancement of their product performance, it is gradually becoming feasible to leverage intelligent devices for health monitoring and management of older adults at the household level. Intelligent devices, characterized by portability, durability, ease of operation, and interactivity, play a significant role in disease prevention, early intervention, provision of efficient diagnosis and treatment services, evaluation of treatment outcomes, and rehabilitation management through health monitoring. This contributes to improving the health status of older adults^4^. Existing research indicates that intelligent development has a positive impact on enhancing the health status of older adults^5,6^. Therefore, can the utilization of intelligent devices in the lives of the elderly generate a broader technological diffusion effect? Especially at the current stage, the excessive growth of household medical expenses has become a significant factor restricting the quality of life for elderly households. Can the use of intelligent devices have a positive effect on reducing household medical consumption expenditures? What is the underlying mechanism? This paper will focus on studying the above questions.

Based on this, this paper utilizes the 2020 China Longitudinal Aging Social Survey (CLASS) data to conduct a thorough exploration of the impact of older adults’ utilization of intelligent devices on their household medical expenditure through theoretical analysis and empirical testing, further examining the underlying mechanisms of this impact. Our research is conducive to providing valuable decision-making reference for the Chinese government to actively respond to population aging, further implement the health-prioritized development strategy with “prevention as the mainstay”, and stimulate the positive role of digital and intelligent empowerment in controlling the rapid growth of medical expenditure. Overall, the marginal contributions of this study are manifested in the following three aspects: Firstly, previous scholars have primarily focused on macro-level perspectives in their research, while this paper delves into the micro-level analysis of the impact of older adults’ utilization of intelligent devices on household medical expenditure, enriching the micro-perspective in medical expenditure research. Secondly, past studies have primarily emphasized the overall impact when analyzing the influencing factors of medical expenditure. This paper not only analyzes the overall impact of older adults’ use of intelligent devices on household medical expenditure but also further explores the micro-level differences in the impact of older adults’ use of intelligent devices on household medical expenditure. Thirdly, unlike previous studies that primarily focused on the mechanism of influence between the two, this paper employs a quantile regression model to deeply explore the developmental patterns of the impact of older adults’ use of intelligent devices on household medical expenditure at different levels.

## Literature review and research hypotheses

### Intelligent devices utilization and household medical expenditure

At the present stage, the rapid progress of digital technologies such as artificial intelligence, the Internet of Things, and big data, and the vigorous development of the digital economy have profoundly influenced various aspects of our lives. For example, some scholars have found through research that the progress of digital technologies or the development of digital intelligence can have significant positive impacts on employment and entrepreneurship^7^ participation in the credit market^8^ improvement of health levels^9^ and the remodeling of household consumption patterns and the upgrading of consumption structures^10,11^. However, at present, the relevant research on how digital development, especially the innovative utilization of intelligent devices, affects household medical consumption expenditure is still limited. In related research, Liu and Li^12^ pointed out that internet use can significantly reduce healthcare consumption at the household level. However, Gu and Cai^13^ presented an opposing viewpoint, arguing that the increase in internet penetration has significantly boosted healthcare expenditure. Lv and Liu^14^suggested that intelligent payment has played a notable role in promoting medical consumption expenditure for rural residents, but it has shown a reducing effect on the self-paid portion. Zeng et al.^15^ pointed out that the utilization of intelligent technologies such as deep learning can scientifically predict medical expenditure at the micro-individual level, thereby better supporting the development of preventive healthcare. Some scholars also believe that the use of intelligent devices in medical diagnosis and treatment can help health systems save significant costs and reduce the burden on healthcare systems^16,17^. Furthermore, intelligent development primarily reduces medical expenditure through enhancing hospital operational efficiency, implementing early interventions to improve patient health, and decreasing the frequent need for patient visits and hospitalizations^18,19^.Currently, intelligent devices are playing an increasingly significant role in healthcare. They primarily conduct dynamic monitoring of target populations through IoT technology and machine learning practices, exerting a positive impact on reducing medical expenditure^20,21^. Overall, based on the synthesis of existing scholarly research, the impact of intelligent technology utilizations on household medical expenditure can be theoretically underpinned by the Preventive Medical Theory and Health Behavior Theory. The Preventive Medical Theory posits that the intelligent devices utilization can reduce the risk of disease deterioration through dynamic monitoring and early warning or intervention, thereby decreasing potential high medical costs in later stages. This aligns with the fundamental pathway in the Health Capital Accumulation Model, where “preventive investments reduce medical demand.” The Health Behavior Theory further suggests that data feedback from intelligent devices enhances individuals’ health management awareness and inhibits risk factors for chronic diseases through a “cognition-behavior” linkage, thereby reducing passive medical consumption. Accordingly, this paper proposes the following research hypothesis:

#### Hypothesis 1

Utilization of intelligent devices by older adults is beneficial for reducing household medical expenditure.

### Intelligent devices utilization, health behaviors, and household medical expenditure

The widespread adoption of intelligent devices serves as a foundational pillar for achieving high-quality vital sign monitoring and health information processing^22^. Grounded in the Health Belief Model (HBM), the utilization of intelligent devices primarily improves health behaviors through two mechanisms. First, it enhances perceived benefits and self-efficacy among older adults. Tikkanen H et al.^23^ highlight that interaction with intelligent devices stimulates individuals’ agency for self-improvement, which essentially relies on the devices’ real-time monitoring and feedback mechanisms^24^. These functions generate personalized health data, enabling self-diagnosis and timely adjustments to health management strategies. This process allows individuals to visualize tangible outcomes of health interventions (e.g., blood pressure trends, calorie expenditure during exercise), thereby reinforcing their belief in improving health through behavioral changes (i.e., self-efficacy in HBM). For instance, Swan M^25^ posits that when users proactively quantify physical activity metrics and modify their health behaviors, intelligent device data act as “cues to action,” triggering a “cognition-behavior linkage”—users recognize the risk of inadequate exercise (perceived susceptibility) through device data and believe increased physical activity reduces disease threats (perceived benefits), thereby adopting healthier lifestyles and enhancing self-efficacy. Second, intelligent devices reduce perceived barriers and provide standardized behavioral guidance. V Chang et al.^26^ emphasize that these devices serve as critical tools for long-term health management, translating abstract health maintenance into concrete, actionable tasks (e.g., achieving 8,000 daily steps) through continuous dynamic tracking. This approach alleviates individuals’ psychological resistance to adopting health behaviors (perceived barriers)^27^. For older adults, integrating intelligent exercise devices with diversified fitness plans enhances motivation via goal-achievement feedback (e.g., virtual rewards upon reaching step targets) and simulated social support (e.g., exercise leaderboards). These strategies align with the HBM pathway of “self-efficacy-driven behavioral change” and demonstrate how devices act as “environmental cues” to activate passive populations (e.g., sedentary older adults)^28,29^. Despite existing evidence that intelligent devices improve older adults’ health behaviors through HBM’s core mechanisms, whether this pathway effectively influences household healthcare expenditures remains unexplored. The theoretical contribution of this paper lies in empirically examining whether “health behavior improvement” plays a mediating role between “older adults’ intelligent device use” and “household medical expenditure”—grounded in the HBM’s logical chain of “health beliefs → health behaviors → health outcomes”. Specifically, intelligent devices first enhance older adults’ health beliefs (e.g., perceived effectiveness of intelligent devices in disease prevention), prompting them to adopt more proactive health behaviors (e.g., regular exercise, timely lifestyle adjustments). These behavioral changes thereby reduce passive medical consumption caused by disease occurrence or exacerbation. Accordingly, the following research hypothesis is proposed in this paper:

#### Hypothesis 2

The intelligent devices utilization can suppress household medical expenditures among older adults by improving or moderating their health behaviors.

### Intelligent devices utilization, medical services demand, and household medical expenditures

Existing studies demonstrate that artificial intelligence (AI) technology significantly enhances patients’ healthcare capabilities and enriches medical services by advancing smart hospitals and wearable health devices^30^. The Technology Acceptance Model (TAM) offers a theoretical framework to explain this phenomenon: intelligent devices strengthen older adults’ perceived usefulness and perceived ease of use through real-time monitoring (e.g., tracking heart rate and blood pressure) and disease prediction algorithms (e.g., machine learning-driven health risk assessments). Specifically, perceived usefulness reflects older adults’ recognition of devices’ practical value in improving health management efficiency and optimizing clinical decisions^31,32^ while perceived ease of use stems from user-friendly interfaces and intuitive data feedback^33^ jointly fostering adoption intentions and sustained usage. Further, this technology acceptance behavior influences medical consumption through dual pathways. On the one hand, rooted in the “attitude-behavior intention” mechanism of the TAM, it enhances older adults’ trust in intelligent devices, driving them to proactively rely on these devices for health monitoring rather than passively depending on traditional medical services^34^. This shift reduces the frequency of unnecessary clinical visits^35^. Second, personalized health data generated by devices (e.g., sleep quality analysis, abnormal symptom alerts) enhance health self-efficacy, encouraging preventive behaviors (e.g., dietary adjustments, regular exercise) to mitigate acute health risks—a process consistent with TAM’s proposition that perceived usefulness positively moderates behavioral outcomes. However, prior research lacks systematic analysis of how intelligent devices reshape individual healthcare demand structures (e.g., shifting from treatment-oriented to prevention-oriented care) and ultimately affect household medical expenditures through TAM. Building on TAM’s “technology features → usage behavior → outcome variables” logical framework, this paper proposes a chained mediation mechanism of “perceived ease of use → usage intensity → demand substitution effect”. We posit that intelligent device utilizations not only directly curb medical expenditures by enhancing health management efficiency at the technological level (e.g., automated data integration and predictive analytics) but also reshape older adults’ healthcare demand preferences at the behavioral decision-making level. Specifically, technology adopters, guided by real-time health feedback (e.g., early symptom detection and personalized risk alerts), shift their preferences from high-cost reactive treatments (e.g., hospitalization for advanced conditions) to low-cost preventive care (e.g., preemptive lifestyle modifications, telehealth follow-ups), thereby mitigating the overexpansion of household medical expenditures. Accordingly, the following research hypothesis is proposed in this paper:

#### Hypothesis 3

The intelligent devices utilization reduces household medical expenditures among older adults by suppressing or moderating their healthcare service demand.

Based on the existing research by scholars, it is evident that the current literature has analyzed the relationship between the use of intelligent devices and medical expenditures, but there are still several research gaps: Firstly, on an overall basis, the existing literature has conducted extensive research on how the use of intelligent devices affects medical expenditures, but the research conclusions are not yet unified. Few scholars have focused on the deep-level relationship between the use of intelligent devices and micro-level household medical expenses. Secondly, scholars’ research suggests that the use of intelligent devices can positively improve the health behaviors of older adults. However, there is currently no further examination of the mechanism of influence between them. Thirdly, previous research has indicated that the use of intelligent devices can enhance individuals’ healthcare capabilities, reduce unnecessary waste of medical resources, and thereby achieve beneficial control over medical expenses. However, existing research has not delved into the mechanism of the role of medical service demand.

**Fig. 1:**
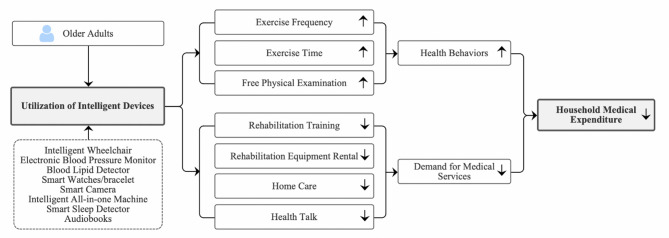
Framework diagram of the influence mechanisms.

The contributions of this paper to the existing literature are as follows: Firstly, on the basis of overall analysis, this paper further investigates the differentiated outcomes of older adults’ use of intelligent devices on household medical expenditures, providing a reference for drawing more refined research conclusions. Secondly, this paper empirically tests and theoretically analyzes whether health behaviors play an effective mechanism of action between older adults’ use of intelligent devices and household medical expenditures. Thirdly, unlike the existing literature, this paper focuses on verifying and analyzing the mediating and moderating effects of medical service demand between the utilization of intelligent devices and micro-level household medical expenses. Based on this, this paper constructs a mechanism framework diagram illustrating the impact of older adults’ use of intelligent devices on household medical expenditures, as shown in Fig. 1.

## Data, variables, and models

### Data source

The data in this paper are derived from the “China Longitudinal Aging Social Survey (CLASS)” jointly released by the Population and Development Research Center and the Institute of Gerontology at Renmin University of China. This project has been conducted biennially since 2014, primarily using a stratified multi-stage probability sampling method, covering 30 provinces, more than 400 village-level units, and over 10,000 older adults aged 60 and above across the country. The CLASS data exhibits strong national representativeness and incorporates information on the usage of intelligent devices by older adults, providing reliable micro-data support for analyzing how the utilization of intelligent devices affects household medical expenditure. This paper utilizes the latest data released in 2020 for research. The CLASS database initially had a total data value of 11,398. After eliminating 1,680 missing values, the final sample size obtained was 9718.

### Variables

#### Dependent variable

The dependent variable studied in this paper is household medical expenditure. Referring to the research by Li and Che^36^ we use the proportion of household medical expenditure to monthly average household expenditure in the past year as the main measurement variable. With economic and social development as well as the improvement of living standards, medical expenditure is bound to show an upward trend year by year. The proportion of medical expenditure to household expenditure is a better indicator reflecting the impact of medical expenditure on family life. Both medical expenditure and monthly average expenditure being logarithmically transformed. The choice of using older adults’ self-reported medical expenditure data in this paper is driven by two key considerations. First, compared to the indirect representation of medical data in administrative records, self-reported data more directly reflect older adults’ decision-making processes regarding medical behaviors. Second, self-reported data are widely used in similar micro-survey studies, establishing a robust foundation for system analysis and theoretical generalization.

#### Explanatory variable

The explanatory variable in this paper is the utilization of intelligent devices, primarily measured by the usage of intelligent devices among older adults. Specifically, the CLASS survey asked older adults respondents about their ownership of intelligent wheelchairs, electronic blood pressure monitors, blood lipid testers, intelligent bracelets/watches, intelligent cameras (with remote monitoring capabilities), intelligent all-in-one devices (such as Baidu’s Xiaodu, Xiaomi’s Xiaoai, etc.), intelligent sleep monitors, and audio books. In this paper, the use of one or more intelligent devices by older adults is defined as 1, otherwise as 0.

#### Control variables

In selecting control variables, based on the analytical framework of this paper and drawing on existing related research^37,38^ this paper chooses the following demographic and socioeconomic characteristics as control variables: (1) individual characteristic variables, specifically including age, gender, education level, marital status, household registration, and smoking status. (2) family characteristic variables, including the number of co-residing family members and the number of properties owned. (3) social characteristic variables, covering participation in the basic social pension insurance and whether individuals continue to engage in income-generating work or activities. (4) regional characteristic variables, including the eastern, central, and northeastern regions, with the western region as the reference group. The definition of the main variables in this paper and their descriptive statistical results are shown in Table 1.

#### Instrumental variables

Regarding the selection of instrumental variables, drawing on the research by Ning and Ma^39^ this paper selects “intelligent device utilization at the district/county level” from the survey sample points as the instrumental variable for intelligent device utilization among older population. Furthermore, based on the research by Lv et al.^40^, “digital coverage level at the district/county level” from the survey sample points is further selected as an instrumental variable for robustness testing. Specifically, intelligent devices utilization at the district/county level is measured by the mean number of intelligent devices owned by older adults at the district/county level. The digital coverage level at the district/county level is measured by the mean digital coverage level of older adults at the district/county level, which corresponds to the question in the questionnaire: “Does your current residence have a network signal (wired or wireless)?“.

#### Mechanism variables

This paper mainly explores the mechanism by which the utilization of intelligent devices influences household medical expenditure from two dimensions: health behavior and healthcare service demand. Specifically, health behavior encompasses three aspects: (1) the frequency of your participation in physical exercise. (2) the average duration of each physical exercise session. (3) whether you have undergone a free physical examination in the past 12 months. The responses to the first and second questions are continuous variables analyzed using the OLS model, while the response to the third question is a binary variable tested using the probit model. Healthcare service demand includes four aspects: whether you require the community medical institution to provide such services, which encompass rehabilitation training, rental of rehabilitation equipment, home nursing, and health seminars. These aspects are primarily analyzed using the probit model.

### Models

#### The two-stage least squares (2SLS) model

The dependent variable in this paper is the medical expenditure of the households where the interviewed older adults reside, which is a typical continuous variable. Therefore, Ordinary Least Squares (OLS) regression is employed for benchmark regression. The set OLS regression equation is as follows:

1 $$\:{MC}_{i}={\alpha\:}_{1}+{\alpha\:}_{2}{IDU}_{i}+{\alpha\:}_{3}{X}_{i}{+\delta\:}_{i}$$

In Eq. (1), $\:{MC}_{i}\:$represents household medical expenditure, $\:{IDU}_{i}$ indicates the utilization of intelligent devices by the interviewed older adults, specifically, the possession of intelligent devices by older adults. $\:{X}_{i}$ represents the control variables, $\:{\delta\:}_{i}$ is the random error term, $\:{\alpha\:}_{1}$is the constant term, and $\:{\alpha\:}_{2}$、$\:{\alpha\:}_{3}$respectively represent the parameters to be estimated for the explanatory variable $\:{IDU}_{i}$ and the control variable$\:{X}_{i}$.

Secondly, due to potential endogenous issues such as omitted variables and reverse causality, ordinary OLS regression may suffer from estimation bias to some extent. Therefore, this paper adopts the Two-Stage Least Squares (2SLS) method to correct for endogeneity, further enhancing the robustness of the estimation results. Regarding the selection of instrumental variables, following the research by Ning and Ma (2018), this paper chooses “intelligent devices utilization at the district/county level” from the survey sample as an instrumental variable for intelligent device utilization among older adults. Furthermore, drawing on the research by Lv et al.^40^, the “digital coverage level at the district/county level” from the survey sample is further selected as an instrumental variable for robustness testing. In terms of the endogeneity of the instrumental variable, districts and counties, as the social environments where the elderly reside, directly increase older adults’ opportunities to access and use intelligent technologies through the prevalence of intelligent devices and the coverage of digital services. For example, the widespread deployment of intelligent medical devices, the popularity of intelligent home systems, and the availability of digital public services within districts and counties all promote the acceptance and use of intelligent devices among the elderly, thereby establishing a positive correlation between the instrumental variable and the explanatory variable. Regarding the exogeneity of the instrumental variable, on the one hand, from the perspective of distinguishing between macro and micro effects, the utilization of intelligent devices and the level of digital coverage at the district/county level may influence overall medical expenses by improving healthcare service efficiency and promoting health management. However, this influence is relatively dispersed and indirect at the individual level, making it unlikely to directly and significantly alter the medical expenditure behaviors of specific elderly individuals or households, thereby preserving the exogeneity of the instrumental variable. On the other hand, from the perspective of policy and environmental factors, the utilization of intelligent devices and the level of digital coverage at the district/county level are primarily determined by macro factors such as local government policies, economic development levels, and infrastructure construction. These factors are generally independent of the medical expenditure decisions of elderly individuals or households, further supporting the exogeneity assumption of the instrumental variable. Additionally, the extent of intelligent devices utilization or digital coverage at the district/county level is measured based on higher-level data^41^. Although the diffusion effect of technology may impact medical expenditures in the regions where elderly populations reside, this impact is relatively small for individual micro-level individuals or households. Therefore, it is assumed that the exogeneity of the instrumental variables holds.The form of the 2SLS model set up in this paper is as follows:

2 $$\:{IDU}_{i}={\beta\:}_{1}+{\beta\:}_{2}{V}_{i}+{\beta\:}_{3}{X}_{i}+{\epsilon\:}_{i}$$

3 $$\:{MC}_{i}={\alpha\:}_{1}+{\alpha\:}_{2}{IDU}_{i}+{\alpha\:}_{3}{X}_{i}+{\delta\:}_{i}$$

Among them, Eq. (2) represents the first-stage regression of the 2SLS model, where $\:{V}_{i}$ denotes the instrumental variable, $\:{X}_{i}$ represents the corresponding control variable, $\:{\beta\:}_{2}$ and $\:{\beta\:}_{3}$ are the parameters to be estimated for the instrumental variable and control variable, respectively, and, $\:{\beta\:}_{1}$ is the constant term. Equation (3) represents the second-stage regression of the 2SLS model, with the same setup as Eq. (1). It primarily estimates the parameter $\:{\alpha\:}_{2}$, which influences$\:{\:MC}_{i}$, using the$\:\:{IDU}_{i}$ obtained from the first-stage regression.

#### Propensity score matching (PSM) model

Since the utilization of intelligent devices among older adults does not exhibit strict exogenous characteristics and is not entirely determined by random factors, older adult’s inherent endowments and family economic conditions significantly influence whether they own intelligent devices. In other words, whether older adults adopt intelligent devices may be a result of their self-selection. For instance, older adults who actively embrace new ideas, are courageous in trying new things, and possess favorable personal or family economic conditions are likely to purchase various intelligent devices, significantly increasing their probability of using such devices. Therefore, whether older adults own intelligent devices is not strictly an exogenous variable but an endogenous dummy variable. Under such premises, using the general OLS regression model to estimate the impact of intelligent device usage on their household medical expenses may result in biased or inconsistent estimation outcomes. In light of this, this paper employs the Propensity Score Matching (PSM) method to address the potential self-selection issue that may arise. Ordinary Least Squares (OLS) regression can only observe the impact of older adults who own intelligent devices on their household medical expenses. However, for older adults without intelligent devices, assuming this group also chooses to purchase intelligent devices, which is the “counterfactual” inference in PSM, we compare this group with the elderly population that uses intelligent devices to obtain a more consistent and pure Average Treatment Effect on the Treated (ATT). The specific calculation method is as follows:

4 $$\:ATT=E\left({Y}_{1i}|{D}_{i}=1\right)-E\left({Y}_{0i}|{D}_{i}=1\right)$$

In the above equation, $\:{Y}_{1i}$ represents the household medical expenditure when older adults adopt intelligent devices, while $\:{Y}_{0i}$ represents the hypothetical household medical expenditure in the control group if older adults had not adopted intelligent devices. Since $\:{Y}_{0i}$ in the control group is not easily observed directly, it is generally necessary to estimate it by constructing a counterfactual framework, which verifies the counterfactual effect $\:\text{E}\left({Y}_{0i}|{D}_{i}=1\right)$ in the estimation of Average Treatment Effect on the Treated (ATT).

#### Quantile regression model

Drawing inspiration from the research design of Wang and Zhao^42^ this paper constructs a quantile regression model to further explore the patterns of influence of older adults ' utilization of intelligent devices on their household medical expenditure. The fundamental idea of quantile regression is as follows: assuming that the explained variable is a random continuous variable, it fits the conditional distribution of the explained variable with the explanatory variables to form a linear function, and ultimately estimates the differences in the regression results of the explanatory variables on the explained variable at different quantile points. The basic form of quantile regression is:

5 $$Q_{\vartheta } Y_{i} /X_{i} = \varphi \vartheta X_{i} ,~\vartheta \in \left( {0,~1} \right)$$

In Eq. (5), $Q_{\vartheta } Y_{i} /X_{i}$ represents the conditional quantile of household medical expenditure $\:{X}_{i}$ influenced by the predetermined vector $\:{Y}_{i}$ consisting of explanatory variables related to older adults’ utilization of intelligent devices. Meanwhile, the method of minimizing the sum of absolute residuals can be employed to estimate the coefficient value$\:\:{\upphi\:}{\upvartheta\:}$ of the equation at the $\:{\upvartheta\:}$ quantile, i.e.,

6 $$\:\:\:\phi\:\vartheta\:={min}\left\{{\sum\:}_{i:{Y}_{i<\phi\:\vartheta\:{X}_{i}}}\vartheta\:\left|{Y}_{i}-\phi\:\vartheta\:{X}_{i}\right|\right.+\left.{\sum\:}_{i:{Y}_{i<\phi\:\vartheta\:{X}_{i}}}(1-\vartheta\:)\left|{Y}_{i}-\phi\:\vartheta\:{X}_{i}\right|\right\}$$.

In Eq. (6), $\:{\upvartheta\:}$ represents the specific value of the quantile point in the quantile regression model, indicating the weight at that quantile point. When the residual is positive, the distribution of sample points lies above the regression point, and the quantile point is $\:{\upvartheta\:}$. Conversely, when the residual is negative, the distribution of sample points is below the regression point, and the quantile point is $\:(1-\vartheta\:)$. As the quantile point $\:{\upvartheta\:}$ gradually approaches 1 from 0, quantile regression can estimate the entire conditional distribution of the impact of older adults’ utilization of intelligent devices, as explanatory variables, on their household medical expenditure.

## Results

### Descriptive statistical analysis

Table 1 reports the definitions of the main variables and their descriptive statistical results. In terms of explained variable, the mean value of household medical expenditure is 0.165, indicating that the proportion of medical expenses for older adults in household expenditure is relatively small, and the household expenditure structure is relatively reasonable. In terms of explanatory variable, the mean value of older adults’ utilization of intelligent devices is 0.423, suggesting that with the vigorous development of the digital economy, intelligent devices such as intelligent wheelchairs, intelligent watches/bracelets are popular among older adults. As for control variables, the average age of older adults is 71.555 years old. Male elderly account for approximately 50.1%. The average educational level of older adults is relatively low, basically at the primary school level. The proportion of married older adults with spouses is 75.8%. The proportion of rural older adults is 48.3%. The proportion of older adults who smoke is 28.2%. The number of family members living with older adults is 2.651. The number of houses owned by the family and the proportion of older adults participating in social pension insurance are 1.074 and 89.8% respectively. In addition, the descriptive statistical results of regional variables, instrumental variables, and mechanism variables are shown in Table 1, which basically meet the research needs.

**Table 1:** Results of descriptive statistics.

Variable		Variable definition	Mean	SD
Explained variable	Household medical expenditure	The proportion of household medical expenses to the average monthly expenditure	0.165	0.166
Explanatory variable	Intelligent devices utilization	Use of one or more intelligent devices = 1; Other = 0	0.423	0.494
Control variables	Age	Actual age of the interviewed	71.555	6.656
	Gender	Male = 1; Female = 0	0.501	0.500
	Educational level	Illiteracy = 0; Literacy/private school = 1; Primary school = 2; Junior high = 3; High school/technical secondary school = 4; College = 5; Bachelor’s degree or above = 6	2.023	1.347
	Marital status	Married with spouse = 1; Other = 0	0.758	0.428
	Household registration status	Non-agricultural household registration = 1; Agricultural registration = 0	0.483	0.500
	Smoking status	Smoking = 1; Non-smoking = 0	0.282	0.450
	Family size	Number of family members	2.651	1.289
	Number of properties	Number of household properties	1.074	0.392
	Social endowment insurance	Participation = 1; No participation = 0	0.808	0.394
	Working status	Having a job = 1; Other = 0	0.253	0.435
Regional variable	Eastern region	Eastern region = 1; Other = 0	0.380	0.485
	Central region	Central region = 1; Other = 0	0.330	0.470
	Western region	Western region = 1; Other = 0	0.253	0.435
	Northeast region	Northeast region = 1; Other = 0	0.123	0.329
Instrumental variables	Utilization of intelligent devices at the district and county level	The average number of intelligent devices owned by older adults at the district and county level	1.091	0.596
	Level of digital coverage at the district and county level	The average digital coverage level of older adults at the district and county level	0.486	0.268
Mechanism variables	Health behaviors	Exercise frequency	3.545	3.419
		Exercise time	1.343	0.659
		Free physical examination	0.561	0.496
	Demand for medical services	Rehabilitation training	0.099	0.299
		Rehabilitation equipment rental	0.093	0.290
		Home care	0.129	0.335
		Health talk	0.242	0.428

Keep three digits after rounding the decimal point.

### Benchmark regression analysis

Table 2 reports the benchmark regression results of the impact of intelligent devices utilization by older adults on household medical expenditure. The results of model (1) indicate that the utilization of intelligent devices by older adults can significantly inhibit the growth of household medical expenditure at the 1% level. In models (2) to (5), a stepwise regression method was adopted, and individual characteristics, family characteristics, social characteristics, and regional characteristic variables were added sequentially. After adding these variables, the utilization of intelligent devices by older adults still significantly reduced the medical expenditure of their households at the 1% level, with a relatively stable coefficient size showing a gradually weakening trend. This suggests that the model performs more robustly after mitigating the issue of omitted variables.

**Table 2:** Results of benchmark regression.

Variables	Household medical expenditure				
	(1)	(2)	(3)	(4)	(5)
Intelligent devices utilization	−0.015^***^(0.003)	−0.013^***^ (0.003)	−0.011^***^ (0.003)	−0.012^***^ (0.003)	−0.010^***^ (0.003)
Individual characteristics					
Age		0.002^***^ (0.0003)	0.002^***^ (0.0002)	0.002^***^ (0.0003)	0.002^***^ (0.0003)
Gender		0.001(0.004)	0.001 (0.004)	0.002 (0.004)	0.002 (0.004)
Educational level		−0.007^***^ (0.001)	−0.006^***^(0.001)	−0.007^***^ (0.001)	−0.007^***^ (0.001)
Marital status		−0.001(0.005)	0.001 (0.005)	0.001 (0.005)	0.001 (0.005)
Household registration status		−0.017^***^ (0.004)	−0.012^***^ (0.004)	−0.022^***^ (0.004)	−0.018^***^ (0.004)
Smoking status		0.009^**^ (0.004)	0.009^**^ (0.004)	0.009^**^ (0.004)	0.009^**^ (0.004)
Family characteristics					
Family size			−0.003^*^ (0.001)	−0.002^*^ (0.001)	−0.002 (0.001)
Number of properties			−0.034^***^ (0.005)	−0.034^***^ (0.005)	−0.028^***^ (0.005)
Social characteristics					
Social endowment insurance				−0.010^**^ (0.004)	−0.009^**^ (0.004)
Working status				−0.035^***^ (0.004)	−0.040^**^ (0.004)
Regional characteristics: western region as reference					
Eastern region					−0.025^***^ (0.004)
Central region					0.007(0.005)
Northeast region					0.012(0.008)
Cons_	0.171^***^ (0.002)	0.035^*^ (0.020)	0.072^***^ (0.021)	0.115^***^ (0.021)	0.115^***^ (0.022)
R^2^	0.002	0.022	0.029	0.036	0.044
N	9718	9718	9718	9718	9718

The values in brackets are robust standard errors, * *p* < 0.1, ** *p* < 0.05, *** *p* < 0.01.

### Robustness test

Table 3 reports the results of the robustness test. This paper adopts two methods: firstly, replacing the explanatory variable “whether intelligent devices are utilized” with “the number of intelligent devices utilized” for estimation; secondly, replacing the explained variable “the proportion of medical expenditure in household expenditure” with “the proportion of medical expenditure in household income” and incorporating it into the model for regression analysis. Both Model (1) and Model (2) indicate that the utilization of intelligent devices by older adults has a significant reducing effect on household medical expenditure, which is consistent with the benchmark regression results.

**Table 3:** Results of robustness test.

Variables	Household medical expenditure	Proportion of medical expenditure in household income
	(1)	(2)
Intelligent devices utilization		−0.005^***^ (0.002)
Number of intelligent devices utilized	−0.004^***^ (0.001)	
Other variables	Controlled	Controlled
Cons_	0.115^***^ (0.022)	0.098^***^ (0.011)
R^2^	0.045	0.284
N	9718	4005

The values in brackets are robust standard errors, * *p* < 0.1, ** *p* < 0.05, *** *p* < 0.01.

### Endogenic treatment

#### Endogenous processing of missing variables: instrumental variable method (IV)

This paper investigates the potential presence of omitted variables that may introduce endogeneity. Therefore, based on this, this paper employs the instrumental variable method to address endogeneity. Table 4 reports the results of the endogeneity test. Model (1) presents the estimation results using “the utilization of intelligent devices at the district/county level” as the instrumental variable, while Model (2) presents the estimation results using “the level of digital coverage at the district/county level” as the instrumental variable. Models (3) and (4) are robustness checks where the explanatory variable is changed from whether intelligent devices are applied to the number of intelligent devices applied.

Regarding the test of instrumental variables, taking Model (1) in Table 4 as an example, the endogeneity test of the 2SLS model indicates that the P-value of the Hausman test is 0.0000, significantly rejecting the exogeneity hypothesis of explanatory variables at the 1% level and proving that the utilization of intelligent devices is an endogenous variable. Moreover, the first-stage F-value in the 2SLS model is 761.01, far exceeding the critical value standard, and the " Wald F statistic” is 1276.617, significantly greater than the 10% significance level critical value (10% maximal IV size: 16.38). This suggests that the instrumental variables possess strong explanatory power and do not exhibit obvious weak instrumental variable issues. Similarly, Models (2) to (4) also yield consistent conclusions regarding the test of instrumental variables, indicating the rationality and effectiveness of the selected instrumental variables. Overall, the results in Table 4 demonstrate that the utilization of intelligent devices by older adults can still significantly reduce household medical expenditure at the 1% level, which is consistent with the benchmark regression results and indicates that the benchmark regression conclusions exhibit good robustness.

**Table 4:** Endogeneity treatment: instrumental variable method.

Variables	Instrumental variable method(2SLS)			
	(1)	(2)	(3)	(4)
The first stage	Intelligent devices utilization		Number of intelligent devices utilized	
Utilization of intelligent devices at the district and county level	0.262^***^ (0.007)		0.978^***^ (0.025)	
Level of digital coverage at the district and county level		0.205^***^ (0.022)		0.589^***^ (0.065)
Stage 1: F value	114.85	21.65	130.32	22.65
R^2^	0.113	0.030	0.170	0.034
Hausman test	0.000	0.000	0.000	0.000
Hansen J statistic	0.000	0.000	0.000	0.000
The second stage	Household medical expenditure			
Intelligent devices utilization	−0.075^***^ (0.008)	−0.150^***^ (0.036)	−0.020^***^ (0.002)	−0.052^***^ (0.012)
Other variables	Controlled	Controlled	Controlled	Controlled
Cons_	0.124^***^ (0.022)	0.135^***^ (0.024)	0.120^***^ (0.022)	0.131^***^ (0.024)
Wald chi2	459.650	413.470	467.020	409.380
R^2^	0.007	0.176	0.025	0.177
N	9718	9718	9718	9718

The values in brackets are robust standard errors, * *p* < 0.1, ** *p* < 0.05, *** *p* < 0.01.

#### Self-selection processing: propensity score matching (PSM)

**Fig. 2:**
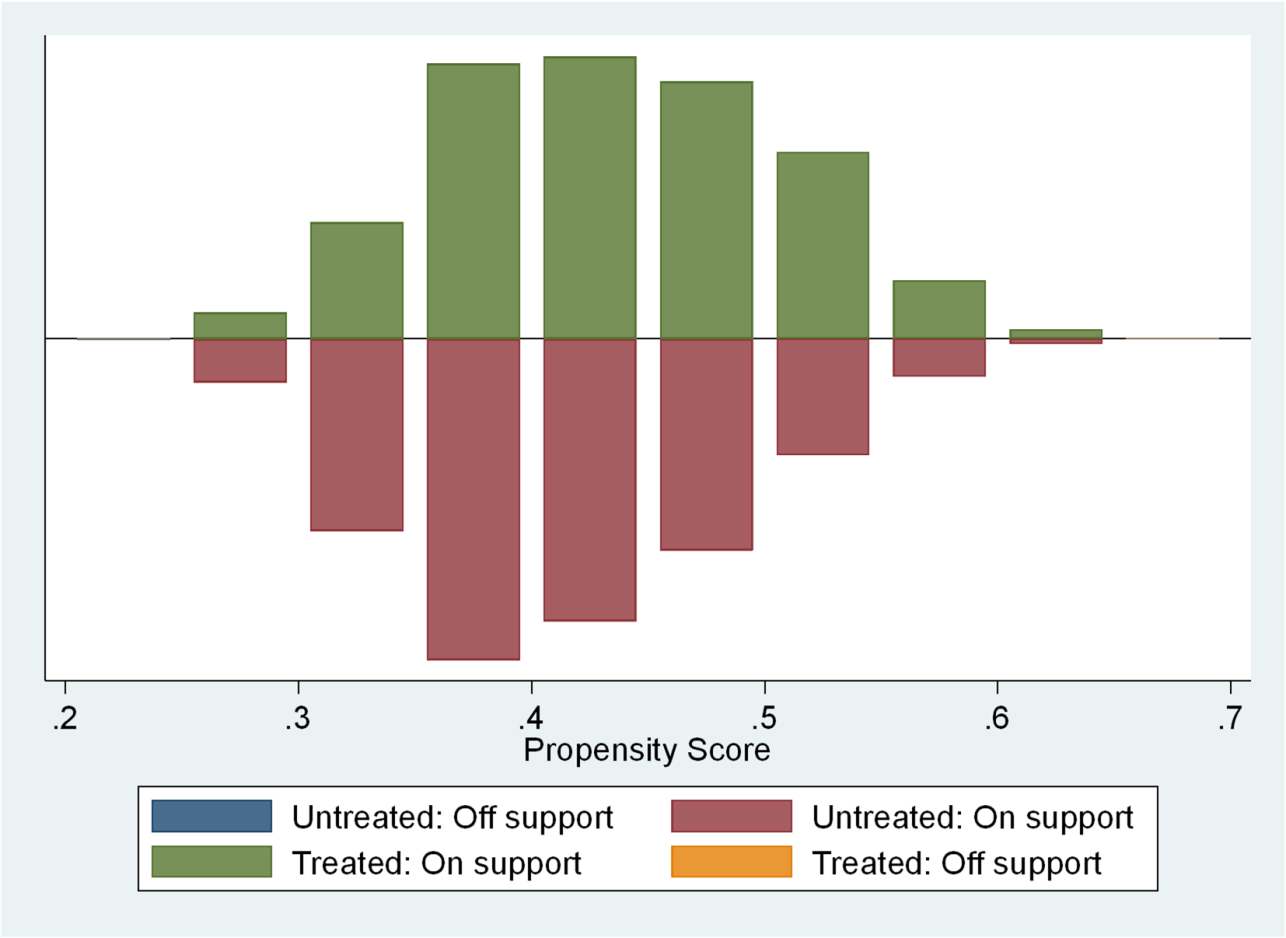
Common value range of propensity score matching.

To control for the issue of self-selection bias among the elderly respondents and delve into the net effect of their utilization of intelligent devices on household medical expenditures, this paper employs the Propensity Score Matching (PSM) method for further estimation. To ensure the quality of PSM estimation, it is necessary to conduct a balancing test first, in order to investigate whether the matching results can effectively balance the distribution of relevant control variables. The results of the balancing test indicate that the matched sample performs well, not only reducing the standard deviation of each variable to below 10% but also rendering most P-values of variables insignificant after matching. As shown in Table 5. This suggests that through PSM, the selective bias in the sample can be largely eliminated, making PSM a reliable method to address the existing selective bias in the sample. Figure 2 illustrates the common support range of PSM, indicating a good state of sample balance and the substantial elimination of sample selection bias.

**Table 5:** Results of the PSM balance test.

Variable	Unmatched	Mean		%bias	%reduct	T-value	P-value
	Matched	Treated	Control		|bias|		
Age	U	71.695	71.453	3.6		1.77	0.077
	M	71.689	71.508	2.7	24.8	1.22	0.222
Gender	U	0.512	0.493	3.8		1.84	0.066
	M	0.512	0.520	−1.5	59.4	−0.70	0.486
Educational level	U	2.111	1.959	11.3		5.48	0.000
	M	2.110	2.136	−2.0	82.5	-0.90	0.370
Marital status	U	0.769	0.751	4.1		1.98	0.047
	M	0.768	0.785	−3.9	3.3	−1.83	0.067
Household registration status	U	0.535	0.445	18.5		8.76	0.000
	M	0.535	0.536	-0.2	98.8	−0.09	0.925
Smoking status	U	0.286	0.278	1.8		0.87	0.385
	M	0.286	0.266	4.4	−148.2	2.02	0.043
Family size	U	2.683	2.629	4.2		2.03	0.042
	M	2.682	2.621	4.8	−14.3	2.18	0.029
Number of properties	U	1.108	1.049	15.0		7.32	0.000
	M	1.108	1.107	0.3	98.2	0.12	0.908
Social endowment insurance	U	0.826	0.795	8.0		3.87	0.000
	M	0.826	0.826	−0.1	98.7	−0.05	0.962
Working status	U	0.212	0.282	−16.3		−7.89	0.000
	M	0.212	0.210	0.5	97.0	0.24	0.814
Eastern region	U	0.438	0.338	20.7		10.12	0.000
	M	0.438	0.437	0.0	99.8	0.02	0.984
Central region	U	0.295	0.356	−13.0		−6.30	0.000
	M	0.295	0.300	−1.0	92.6	−0.45	0.654
Northeast region	U	0.100	0.140	−12.4		−5.96	0.000
	M	0.100	0.102	−0.5	95.9	−0.25	0.806

Table 6 reports the results of the propensity score matching (PSM). This paper primarily employs three commonly used PSM methods for empirical testing: nearest neighbor matching, caliper matching, local linear regression matching, and kernel matching. The focus is on estimating the average treatment effect on the treated (ATT) between the experimental group and the control group regarding the use of intelligent devices by older adults. In the specific testing process, we adopt a replacement matching approach, giving priority to basic demographic attributes such as age and gender, to avoid these characteristics interfering with the specific estimation results of the propensity score matching. The estimation results of nearest neighbor matching indicate that the ATT value of the impact of intelligent devices usage on household medical expenditures by older adults is -0.015 before matching, but it becomes − 0.007 after matching. The results of caliper matching are consistent with this, and both are significant at the 10% level. For local linear regression matching, the ATT value after matching is -0.008, which is significant at the 5% level. Regarding kernel matching, the ATT value after matching is -0.010, also significant at the 1% level. These results suggest that the net effect of intelligent devices usage by older adults on household medical expenditures decreases by 0.7%, 0.7%, 0.8%, and 1% respectively. Thus, it can be seen that the impact of intelligent devices usage by older adults on household medical consumption expenditures exhibits good robustness after estimation using the PSM method. If the selective bias issue in this study is not addressed, it may overestimate the reduction effect of intelligent devices usage on household medical expenditures to some extent.

**Table 6:** Self-selected processing: PSM model testing.

Variables and matching methods	Category	Treatment group	Control group	ATT	Standard error	T-value
Intelligent devices utilization	Before matching	0.156	0.171	−0.015	0.003	−4.33^***^
Nearest neighbor matching	After matching	0.156	0.164	−0.007	0.004	−1.91^*^
Caliper matching	After matching	0.156	0.164	−0.007	0.004	−1.93^*^
Local linear regression matching	After matching	0.156	0.164	−0.008	0.004	−1.98^**^
Nuclear matching	After matching	0.156	0.167	−0.010	0.003	−3.13^***^

* *p* < 0.1, ** *p* < 0.05, *** *p* < 0.01. The nearest neighbor matching adopts 1 to 4 matching, and the radius value of caliper matching is 0.05.

### Heterogeneity analysis

#### Microscopic heterogeneity analysis

Older adults are not entirely homogeneous, and the impact of intelligent devices utilization on household medical expenditure among older adults with different characteristics exhibits notable heterogeneity. Based on this observation, this paper primarily analyzes the micro-level differences in this impact from the perspectives of age, gender, number of cohabitating family members, household registration, education level, and retirement status. Table 7 reports the estimation results. Models (1) to (6) respectively incorporate interaction terms between intelligent devices utilization and age, gender, number of cohabitating family members, household registration, education level, and retirement status. The estimated partial regression coefficients of the interaction terms indicate that the influence of intelligent devices utilization on household medical expenditure among older adults varies significantly across different factors, except for gender. Specifically, for older adults and those living with family members, intelligent devices utilization significantly reduces the growth of household medical expenditure at the 1% and 5% levels, respectively. However, for older adults with urban household registration, those with a junior high school education or above, and retired individuals, intelligent devices utilization increases household medical expenditure at the 1%, 5%, and 10% levels, respectively.

**Table 7:** Results of microscopic heterogeneity.

Variables	Household medical expenditure					
	(1)	(2)	(3)	(4)	(5)	(6)
Intelligent devices utilization	0.115^***^ (0.035)	−0.008^*^ (0.004)	0.008 (0.007)	−0.019^***^ (0.005)	−0.015^***^ (0.004)	−0.014^***^ (0.004)
Intelligent devices utilization ×Age	−0.002^***^ (0.000)					
Intelligent devices utilization × Gender		-0.004 (0.006)				
Intelligent devices utilization × Family size			−0.007^**^ (0.003)			
Intelligent devices utilization × Household Registration				0.019^***^ (0.007)		
Intelligent devices utilization × Educational level (Junior high school and above)					0.009^**^ (0.004)	
Intelligent devices utilization ×Retirement						0.009^*^ (0.005)
Other variables	Controlled	Controlled	Controlled	Controlled	Controlled	Controlled
Cons_	0.060^**^ (0.029)	0.114^***^ (0.022)	0.106^***^ (0.022)	0.118^***^ (0.022)	0.123^***^ (0.023)	0.117^***^ (0.022)
R^2^	0.045	0.044	0.045	0.045	0.040	0.044
N	9718	9718	9718	9718	8317	9718

The values in brackets are robust standard errors, * *p* < 0.1, ** *p* < 0.05, *** *p* < 0.01.

#### Macroscopic heterogeneity analysis

Considering the varying levels of economic development and digitization across regions where the elderly reside, this paper further analyzes macro-level differences based on different regions and levels of digital coverage. Table 8 reports the estimation results of macro-level heterogeneity. Models (1) to (4) respectively incorporate interaction terms between intelligent devices utilization and the eastern, central, western, and northeastern regions, while Model (5) includes an interaction term between intelligent devices utilization and the level of digital coverage. The empirical results indicate that for the elderly residing in the eastern, western, and high digital coverage regions, intelligent devices utilization can reduce household medical expenditure at the 1% level. However, for older adults in the central and northeastern regions, the utilization of intelligent devices significantly increases household medical expenditure at the 1% level.

**Table 8:** Results of macroscopic heterogeneity.

Variables	Household medical expenditure				
	(1)	(2)	(3)	(4)	(5)
Intelligent devices utilization	−0.004 (0.004)	−0.023^***^ (0.003)	−0.008^**^ (0.003)	−0.017^***^ (0.003)	−0.002 (0.004)
Intelligent devices utilization ×Eastern region	−0.020^***^ (0.004)				
Intelligent devices utilization ×Central region		0.035^***^ (0.005)			
Intelligent devices utilization ×Western region			−0.019^***^ (0.005)		
Intelligent devices utilization ×Northeast region				0.047^***^ (0.009)	
Intelligent devices utilization ×Digital coverage level					−0.017^***^ (0.004)
Other variables	Controlled	Controlled	Controlled	Controlled	Controlled
Cons_	0.115^***^ (0.021)	0.110^***^ (0.021)	0.112^***^ (0.021)	0.112^***^ (0.021)	0.116^***^ (0.022)
R^2^	0.037	0.040	0.037	0.039	0.045
N	9718	9718	9718	9718	9718

The values in brackets are robust standard errors, * *p* < 0.1, ** *p* < 0.05, *** *p* < 0.01.

#### Quantile regression analysis

To delve into the specific impact of intelligent devices utilization among older adults on household medical expenditure, this paper employs quantile regression analysis for further exploration. Specifically, the estimation results at the 10th, 25th, 50th, 75th, and 90th quantiles are selected. The results in Table 9 indicate that at the 10th and 25th quantiles, the utilization of intelligent devices by older adults significantly promotes household medical expenditure at the 1% level, with coefficient values of 0.004 and 0.005, respectively. At the 50th quantile, the utilization of intelligent devices by older adults has a significantly positive effect on household medical expenditure at the 5% level, with a coefficient value of 0.006, indicating a minor change. However, at the 75th and 90th quantiles, the utilization of intelligent devices by older adults significantly reduces household medical expenditure at the 5% and 1% levels, respectively, and the inhibitory effect notably increases from the coefficient values. These findings suggest that the impact of intelligent devices utilization by older adults on household medical expenditure exhibits a clear quantile effect, demonstrating an “inverted U-shaped” trend of initial increase followed by a decrease.

**Table 9:** Results of quantile regression.

Variables	Household medical expenditure				
	Q10	Q25	Q50	Q75	Q90
Intelligent devices utilization	0.005^***^ (0.002)	0.004^***^ (0.002)	0.006^**^ (0.002)	−0.008^**^ (0.004)	−0.037 ^***^ (0.009)
Other variables	Controlled	Controlled	Controlled	Controlled	Controlled
Cons_	−0.023^*^ (0.013)	0.023^**^ (0.011)	0.079^***^ (0.015)	0.149^***^ (0.027)	0.192^***^ (0.062)
R^2^	0.020	0.028	0.027	0.030	0.069
N	9718	9718	9718	9718	9718

The values in brackets are robust standard errors, * *p* < 0.1, ** *p* < 0.05, *** *p* < 0.01.

### Impact mechanism analysis

Previous analysis has shown that the utilization of intelligent devices significantly reduces household medical expenditure, accompanied by heterogeneous and quantile effects. Based on this, to delve deeper into the impact mechanism of intelligent devices utilization by older adults on household medical expenditure, this paper primarily focuses on two dimensions: health behavior and demand for medical services.

Table 10 presents the results of the mediation analysis examining the impact of intelligent device utilization on household medical expenditure, based on two dimensions: health behavior and demand for medical services.

Firstly, Models (1) to (3) present the validation results for health behavior. Models (1) and (2) indicate that the utilization of intelligent devices by older adults significantly promotes their exercise frequency and time at the 10% and 1% levels, respectively. The results of Model (3) show that intelligent devices utilization significantly increases the enthusiasm of older adults to participate in free physical examinations at the 1% level. Overall, the utilization of intelligent devices significantly improves the health behavior of older adults. The Health Belief Model suggests that when individuals perceive behaviors that are harmful to their health or potential threats of illness, they will develop a clear expectation of the dangerous consequences of such perceptions. As individuals with high self-efficacy, they will actively take actions to implement behavioral changes that are beneficial to their health^43^. In light of the research presented in this paper, the utilization of intelligent devices by older adults allows for long-term dynamic monitoring of individual behavior and personal status through scientific and reasonable methods. This enables older adults to have a clearer understanding of whether their behavior is healthy and whether they have potential disease threats, and to promptly adopt health-promoting behavioral changes, such as increasing exercise frequency and duration, and regularly participating in physical examinations. These behavioral changes effectively improve health levels and contribute to reducing household medical consumption expenditure.

Secondly, models (4) to (7) represent the empirical tests of healthcare service demand. The results indicate that the utilization of intelligent devices can significantly reduce the demand for healthcare services such as rehabilitation training, rental of rehabilitation equipment, home care, and health lectures among the elderly population at the 1% level. The reduction in healthcare service demand among older adults can lead to their proactive decrease in the utilization of medical resources, thereby exerting a notable inhibitory effect on household medical expenses. According to the theory of health demand, health is a form of capital, and healthcare services are behaviors that can enhance health capital stock. Factors influencing healthcare service demand include personal, economic, and social factors^44^. With advancing age, the physical functions of the elderly deteriorate, and various diseases become more prevalent, leading to an accelerated decline in health capital. This, in turn, prompts them to have a strong demand for healthcare services to maintain or promote the appreciation of their health capital. The utilization of intelligent devices has a significant reducing effect on the healthcare service demand of older adults. This is because the utilization of intelligent devices is a key factor that can influence the healthcare service demand. It is not only a personal choice behavior of older adults but also closely related to the economic strength of individuals or households. Meanwhile, the utilization of intelligent devices can enhance the scientific understanding of the elderly regarding their health capital and its development patterns through dynamic monitoring of their health status. This reduces the demand for healthcare services such as rehabilitation training, rental of rehabilitation equipment, home care, and health lectures, thereby avoiding unnecessary waste of medical resources and helping to reduce household medical expenses.

**Table 10:** Results of mediation effect analysis.

Variables	Health behaviors			Demand for medical services			
	Exercise frequency	Exercise time	Free physical examination	Rehabilitation training	Rehabilitation equipment rental	Home care	Health lectures
	(1)	(2)	(3)	(4)	(5)	(6)	(7)
Intelligent devices utilization	0.105^*^ (0.063)	0.033^***^ (0.012)	0.077 ^***^ (0.026)	−0.152^***^ (0.038)	−0.185 ^***^ (0.039)	−0.098 ^***^ (0.034)	−0.116^***^ (0.030)
Other variables	Controlled	Controlled	Controlled	Controlled	Controlled	Controlled	Controlled
Cons_	6.248^***^ (0.461)	1.651^**^ (0.070)	−1.022^***^ (0.174)	2.374^***^ (0.253)	1.991^***^ (0.258)	−2.319^***^ (0.225)	−0.957^***^ (0.196)
R^2^	0.091	0.181	0.037	0.030	0.027	0.019	0.035
N	11,398	11,398	9906	9064	9035	9483	9212

The values in brackets are robust standard errors, * *p* < 0.1, ** *p* < 0.05, *** *p* < 0.01.

Tables 11 and 12 present the results of the moderation analysis examining the influence of intelligent devices utilization on household medical expenditures, based on the two dimensions of health behavior and demand for medical services.

Table 11 presents the results of the moderation analysis examining the influence of health behaviors (exercise time, exercise frequency, and free physical examination) on the mechanism through which intelligent devices utilization affects household medical expenditures. The results from Model (1) in Table 11 indicate that the utilization of intelligent devices by the elderly, exercise frequency, and the interaction term between intelligent devices utilization and exercise frequency significantly inhibit household medical expenditures at the 5%, 1%, and 1% levels, respectively, and the effects are in the same direction. The results from Model (2) indicate that the utilization of intelligent devices by the elderly, exercise time, and the interaction term between intelligent devices utilization and exercise time all significantly reduce household medical expenditures at the 1% level, with the effects being in the same direction. The results from Model (3) show that the utilization of intelligent devices by the elderly, free physical examination, and the interaction term between intelligent device utilization and free physical examination significantly decrease household medical expenditures at the 5%, 1%, and 5% levels, respectively, with the effects also being in the same direction. Thus, it can be seen that health behaviors, represented by exercise time, exercise frequency, and free physical examination, all play a significant positive moderating role in reducing household medical expenditures through the utilization of intelligent devices.

**Table 11:** Results of moderating effect analysis (Health behavior).

Variables	Household medical expenditure			
	(1)	(2)		(3)
Intelligent devices utilization	−0.012^**^		−0.026^***^	−0.001^**^
	(0.006)		(0.007)	(0.0005)
Exercise frequency	−0.002^***^			
	(0.001)			
Intelligent devices utilization × Exercise frequency	−0.001^***^			
	(0.0003)			
Exercise time			−0.004^***^	
			(0.001)	
Intelligent devices utilization × Exercise time			−0.012^***^	
			(0.004)	
Free physical examination				−0.012^***^
				(0.005)
Intelligent devices utilization × Free physical examination				−0.016^**^
				(0.007)
Other variables	Controlled		Controlled	Controlled
Cons_	0.102^***^		0.118^***^	0.127^***^
	(0.022)		(0.022)	(0.023)
R^2^	0.046		0.045	0.054
N	9718		9718	8505

The values in brackets are robust standard errors, * *p* < 0.1, ** *p* < 0.05, *** *p* < 0.01.

Table 12 presents the test results of the moderating mechanism through which the utilization of intelligent devices affects household medical expenditure, with regard to the demand for medical services (rehabilitation training, rehabilitation equipment rental, home care, and health lectures). The results of Model (1) in Table 12 show that rehabilitation training significantly increases household medical expenditure at the 1% level. However, the utilization of intelligent devices among the elderly and the interaction term between the utilization of intelligent devices and rehabilitation training significantly suppress household medical expenditure at the 10% and 5% levels respectively, and the directions of their impacts are the same. The results of Model (2) show that the rental of rehabilitation equipment significantly increases household medical expenses at the 1% level. However, the utilization of intelligent devices among the elderly and the interaction term between the utilization of intelligent devices and the rental of rehabilitation equipment both significantly reduce household medical expenses at the 5% level, and the impacts of the two are in the same direction. The results of Model (3) demonstrate that home care significantly increases household medical expenses at the 1% level. However, the utilization of intelligent devices among the elderly and the interaction term between the utilization of intelligent devices and home care significantly reduce household medical expenses at the 5% and 1% levels respectively, and the impacts of the two are in the same direction. The results of Model (4) show that health lectures significantly increase household medical expenses at the 1% level. However, the utilization of intelligent devices among the elderly and the interaction term between the utilization of intelligent devices and health lectures both significantly reduce household medical expenses at the 5% level, and the impacts of the two are in the same direction. It can be seen from the above that the demand for medical services represented by rehabilitation training, the rental of rehabilitation equipment, home care and health lectures can all play a significant positive moderating role in reducing household medical expenses through the utilization of intelligent devices.

**Table 12:** Results of moderating effect analysis (Demand for healthcare services).

Variables	Household medical expenditure			
	(1)	(2)	(3)	(4)
Intelligent devices utilization	−0.007^*^	−0.008^**^	−0.007 ^**^	−0.002^**^
	(0.004)	(0.004)	(0.003)	(0.001)
Rehabilitation training	0.028^***^			
	(0.008)			
Intelligent devices utilization× Rehabilitation training	-0.023^**^			
	(0.011)			
Rehabilitation equipment rental		0.019^***^		
		(0.007)		
Intelligent devices utilization× Rehabilitation equipment rental		−0.002^**^		
		(0.001)		
Home care			0.039^***^	
			(0.008)	
Intelligent devices utilization× Home care			−0.013^***^	
			(0.003)	
Health lectures				0.039^***^
				(0.005)
Intelligent devices utilization× Health lectures				−0.014^**^
				(0.007)
Other variables	Controlled	Controlled	Controlled	Controlled
Cons_	0.124^***^	0.125^***^	0.140^***^	0.124^***^
	(0.025)	(0.025)	(0.024)	(0.024)
R^2^	0.055	0.055	0.057	0.059
N	7826	7814	8188	7904

The values in brackets are robust standard errors, * *p* < 0.1, ** *p* < 0.05, *** *p* < 0.01.

## Discussion

The increasing abundance of intelligent devices presents new opportunities for controlling the rise of medical expenses at the micro-level of households. This paper employs data from the 2020 China Longitudinal Aging Social Survey (CLASS) to empirically analyze the impact of older adults’ utilization of intelligent devices on household medical expenditures and explores the underlying mechanisms. The research findings indicate that the utilization of intelligent devices by older adults significantly reduces household medical expenditures. This conclusion remains valid after robustness checks and endogeneity treatments. This result is generally consistent with the findings of Hung & Lin^45^ and Sahni et al.^18^. The possible reasons for this outcome are twofold. On the one hand, with intelligent devices, older adults can monitor their health status and daily activities in real-time, continuously, and dynamically, facilitating the formation of healthy behavioral habits. Regular interaction with intelligent devices can effectively enhance their health levels and reduce household medical expenditures. On the other hand, the utilization of intelligent devices helps mitigate their indiscriminate use of medical services or overutilization due to “moral hazard” on the patient side. This, in turn, contributes to restraining the unreasonable growth of household medical expenditures to some extent^19^.

The results of this study on heterogeneity indicate that the utilization of intelligent devices has a reducing effect on household medical expenditures for older adults who are older, living with family, residing in eastern and western regions, and in areas with high digital coverage. Additionally, for older adults with urban hukou, an educational level of junior high school or above, who are retired, or living in central and northeastern regions, the utilization of intelligent devices significantly promotes an increase in household medical expenditures. As age increases, physical function deterioration elevates the morbidity rate of older adults, leading to a significant rise in household medical expenditures. The utilization of intelligent devices enhances the health awareness and capabilities of older adults, inhibiting the growth of household medical expenditures^35^. Older adults in regions with high digital coverage find it more convenient to use intelligent devices, which may have a more pronounced impact on household medical consumption expenditures. Older adults who are retired, hold urban hukou, and have an educational level of junior high school or above may engage in excessive medical treatment when using intelligent devices, thereby leading to an increase in household medical expenditures^30^. The eastern region, due to its economic development, has a higher level of medical security and relatively better health status for older adults. When using intelligent devices, they may focus more on disease prevention and healthcare, contributing to the suppression of household medical expenditures^46^. There are fewer older adults using intelligent devices in the western region, and even if health risks are detected by intelligent devices, they may selectively ignore them. Older adults in the central region may be in the initial stage of intelligent devices utilization, while those in the northeastern region, due to severe aging, may not experience a significant suppressive effect of intelligent devices utilization on household medical expenditures^34^. Overall, there are significant regional differences in the impact of intelligent devices utilization on household medical expenditures.

This paper focuses on exploring the trend of the impact of older adults’ utilization of intelligent devices on household medical expenditure by constructing a quantile regression model. The results indicate that there is a significant quantile effect in the impact of older adults’ utilization of intelligent devices on household medical expenditure, exhibiting an “inverted U-shaped” development trend. When older adults initially start using intelligent devices, they tend to pay close attention to various health indicators displayed by these devices and strictly compare them with medical norms. This behavior not only prompts older adults to actively consume various medical resources to restore abnormal indicators to normal levels but also leads to a notable increase in household medical expenditure. Secondly, as the level of intelligent devices utilization continues to rise, older adults gain a more comprehensive understanding of their physical and mental health status. They gradually shift their focus to enhancing health awareness and reducing excessive reliance on professional medical services and resources, thereby decreasing household medical expenditure. Lastly, intelligent devices such as intelligent wheelchairs and blood lipid detectors are expensive and fall under the category of medical equipment. Some families may include them in medical expense budgets, resulting in an increase in household medical expenditure.

Our research employs a combination of empirical analysis and theoretical analysis to delve into the mechanism through which the utilization of intelligent devices by older adults affects household medical expenditure, examining it from two dimensions: health behavior and demand for medical services. The research findings indicate that the utilization of intelligent devices can reduce household medical expenditure through two pathways: improving the health behavior of older adults and decreasing the demand for medical services. This result is consistent with the findings of Ni et al.^4^, Chehri & Mouftah^35^, and Srivastava & Routray^47^, and also provides reasonable validation for Hypotheses 2 and 3.

However, this paper has several limitations requiring deeper exploration in future research:

First, the findings of this paper may be constrained by the socioeconomic characteristics and regional context of the current sample, and thus cannot fully circumvent potential limitations in external validity. However, we intend to conduct integrated analyses based on cross-national or cross-regional data in future studies to further enhance the external validity of this research. On the other hand, individual cities in China are not included in the CLASS data used in this paper and were excluded from our analysis. Fortunately, the volume of excluded data is relatively small, exerting minimal influence on our research findings.

Second, the use of self-reported medical expenditure data as the dependent variable in this paper may introduce minor measurement bias limitations. However, self-reported medical expenditure data also have inherent rationality: in contrast to the indirect representation of administrative records, self-reported data more directly reflect the decision-making processes behind individual medical behaviors. Additionally, self-reported data are widely used in similar micro-survey studies, establishing a robust foundation for behavioral analysis. To address this limitation, future research intends to utilize macro-level healthcare expenditure data from national statistical yearbooks to deeply validate the impact of intelligent device applications on healthcare spending from a macro perspective.

Third, our study has not fully accounted for the dynamic impacts of COVID-19. The dataset utilized here covers the period up to 2020, during which pandemic-related fluctuations exerted minimal influence on the outcomes. We acknowledge this temporal limitation and plan to address COVID-19’s confounding effects through two strategies. First, upon the availability of updated data, we will integrate multi-wave panel data to test the temporal stability of our findings. Second, we will conduct comparative analyses across pre-pandemic, pandemic-onset, and post-pandemic phases to disentangle the net effects of policy interventions from COVID-19-induced variations. This phased approach will enhance the robustness of causal inferences in evolving public health contexts.

## Conclusions and policy recommendations

Using data from the 2020 China Longitudinal Aging Social Survey (CLASS), this paper analyzes the impact of older adults’ utilization of intelligent devices on household medical expenditure. The findings are as follows: (1) The utilization of intelligent devices by older adults significantly reduces household medical expenditure. This conclusion remains valid after placebo tests and endogeneity treatment. (2) Heterogeneity analysis indicates that the utilization of intelligent devices has a pronounced effect on reducing medical expenditure for households with older adults who are relatively older, living with family, residing in eastern and western regions, and in areas with high digital coverage. However, for older adults with urban hukou, an education level of junior high school or above, who are retired, and residing in central and northeastern regions, the use of intelligent devices significantly increases household medical expenditure. (3) Quantile regression results reveal that the impact of older adults’ utilization of intelligent devices on household medical expenditure exhibits an “inverted U-shape” trend, with an initial increase followed by a decrease. (4) Mechanism analysis suggests that the utilization of intelligent devices by older adults reduces household medical expenditure through two pathways: improving health behavior and decreasing the demand for medical services.

Based on the above research conclusions, this paper proposes the following countermeasures and suggestions: Firstly, accelerate the development and utilization of intelligent devices suitable for older adults. Encourage various enterprises and research institutions to develop new intelligent devices tailored to the characteristics of older adults, including functional enhancements and exploration of new functionalities, and continuously improve the level of aging-friendliness of intelligent devices. Secondly, for underdeveloped rural or remote areas, encourage the provision of high price subsidies or significant discounts to older adults purchasing intelligent devices through financial subsidies, in order to enhance the utilization of intelligent devices among vulnerable elderly groups and curb unreasonable increases in household medical expenses. Thirdly, strengthen publicity and education on intelligent devices and disseminate health knowledge to increase the recognition and acceptance of intelligent devices among older adults. Fourthly, promote multi-party collaboration between older adults’ intelligent devices, primary medical institutions, community elderly care institutions, and integrated medical-elderly care institutions. Establish a remote health management system and a timely medical service system for older adults based on the utilization of intelligent devices, continuously improve the health level of older adults, and reduce unnecessary expenditures on household medical expenses.

## Data Availability

Data available in a publicly accessible repository (China Longitudinal Aging Social Survey, CLASS). http://class.ruc.edu.cn/. The Datasets used and analyzed during the current study are available from the corresponding author on reasonable request.
